# Triple‐Osteotomy leads to substantially improved quality of life in patients with hip dysplasia

**DOI:** 10.1002/jeo2.70208

**Published:** 2025-03-07

**Authors:** Julia Elisabeth Lenz, Moritz Riedl, Dominik Szymski, Stefan Landgraeber, Volker Alt, Stefan Fickert

**Affiliations:** ^1^ Department of Trauma Surgery University Hospital Regensburg Regensburg Germany; ^2^ Department of Orthopedics and Orthopedic Surgery Saarland University Medical Center Homburg Germany; ^3^ Sporthopaedicum Straubing Straubing Germany

**Keywords:** Germany, hip, iHOT33, quality of life, triple

## Abstract

**Purpose:**

Triple‐Osteotomy (TO) is a hip‐preserving surgical technique designed to correct symptomatic hip dysplasia by achieving three‐dimensional acetabular reorientation and improving femoral head coverage. This procedure has shown promising outcomes in pain reduction, functional recovery, and quality of life, particularly in young, active patients. While periacetabular‐osteotomy (PAO) is another well‐established method for hip preservation, the specific advantages of TO, especially in early recovery and patient‐reported outcomes (PROMs), remain underexplored. This study evaluates the mid‐term outcomes of TO using the iHOT33 tool to provide a comprehensive understanding of its clinical benefits.

**Methods:**

This non‐randomised, retrospective registry study within the German Cartilage Registry included 48 patients with symptomatic, radiologically confirmed hip dysplasia who underwent TO by the same specialist. The follow‐up rate at 24 months was 60.4% with a mean follow‐up time of 24 months. Outcomes measured included iHOT33 scores, quality of life, VAS for pain, satisfaction, perceived treatment benefit, and unemployment rate. Paired t‐tests and regression analysis (*p* < 0.05) were applied.

**Results:**

Preoperative iHOT33 scores averaged 46.9, increasing to 70.8 after 24 months (Δ 23.9), with notable improvement in the first 6 months (Δ 15.8). The “social” subdomain showed the greatest improvements (Δ 30 points), alongside improvements in quality of life and pain reduction (VAS). Postoperative angles (VCE 31° ± 4°, acetabular index 0° ± 3°) were within the normal range. No significant correlation was found between angle changes and iHOT33 scores, indicating benefits across dysplasia severities.

**Conclusions:**

Triple‐osteotomy offers significant and rapid improvements in patient‐reported outcomes for individuals with hip dysplasia, particularly in enhancing social and sports‐related quality of life as measured by iHOT33 and other subjective assessments. Its potential advantages over Periacetabular‐osteotomy, especially in terms of early recovery, warrant further investigation through prospective, comparative studies to better define its role in hip‐preserving surgical strategies.

**Level of Evidence:**

Level III.

**Abbreviations**
HTE angle: acetabular roof angle
iHOT33: International Hip Outcome Tool 33
LCE angle: lateral centre‐edge angle
MCID: minimal clinically important difference
PAO: periacetabular osteotomy
PASS: patient acceptable symptom state
PROM: patient‐reported outcome measures
SCB: substantial clinical benefit
Triple‐Osteotomy, TO: triple‐pelvic‐osteotomy
VAS: Visual Analog Scale
VCA angle: vertical‐centre‐anterior angle

## INTRODUCTION

Hip dysplasia is characterised by insufficient development of the hip joint, resulting in reduced coverage of the acetabulum, lateralisation of the femoral head, instability, and consecutive damage to labrum and cartilage [6, 8, 15]. Moreover, it is a known risk factor for early osteoarthritis of the hip, underscoring its significant clinical impact on long‐term joint health and functionality. Early and effective intervention is crucial to preserving hip joint function and delaying or preventing osteoarthritis.

Triple‐pelvic‐osteotomy (Triple‐Osteotomy), also known as Tönnis‐Osteotomy named after its founder, is a surgical procedure developed to treat hip dysplasia [19, 24]. It involves the osteotomy of all three pelvic bones (ischium, pubis and ilium) to enable the correction of the acetabulum's position. This procedure has demonstrated positive outcomes in terms of functional scores [3, 4, 21, 22, 23]. However, there is limited literature on the subjective patient‐related outcome following Triple‐Osteotomy, especially regarding quality of life and functional recovery.

Periacetabular‐osteotomy (PAO) is another widely used hip‐preserving procedure for treating hip dysplasia, and several studies have documented its effectiveness in improving patient‐reported outcomes and joint stability [2, 5, 16, 18]. Despite these similarities, the comparative advantages of Triple‐Osteotomy, particularly regarding early recovery and its impact on younger, active patients, remain underexplored.

Patient‐related outcome scores like the “International Hip Outcome Tool 33” (iHOT33) measures pain, function, sport activities and overall quality of life associated at the hip joint in a standardised way [12]. This score is widely used in young, active patients to assess the results of hip surgery, and it has been proven to be reliable, valid, and sensitive to changes in hip‐related quality of life.

Nevertheless, as far as the authors are aware, there is currently no evaluation of the mid‐term effects of Triple‐Osteotomy using subjective patient‐related outcome results.

This study hypothesises that Triple‐Osteotomy provides significant and early improvements in patient‐reported outcome measures (PROMs), particularly in social and sports‐related quality of life, compared to other surgical interventions for hip dysplasia, such as PAO.

This study aims to analyse the mid‐term results of Triple‐Osteotomy using the iHOT33, focusing on patient‐reported outcomes such as pain, function, sports activities, and quality of life. Additionally, it investigates whether clinical outcomes correlate with independent parameters or radiographic corrections.

## MATERIALS AND METHODS

The study is a non‐randomised, non‐controlled, retrospective register study as part of the German Cartilage Registry (KnorpelRegister DGOU), approved by the institutional review board of the University of Freiburg (No. 520/14) and the participating centres itself. The German Cartilage Registry is funded by the German Orthopaedic and Trauma Society (DKOU), the “Deutsche Arthrose‐Hilfe e.V.” and “Oscar‐Helene‐Stiftung e.V.”

### Surgical technique

All patients underwent surgery performed by the same highly experienced surgeon specialised in hip preserving operations. The surgical technique involved osteotomy of the ischium in the lateral position, followed by osteotomy of the pubis and ilium in the supine position. Using image intensification, the acetabulum rotated in three dimensions to optimise the coverage of the femoral head. Stable fixation was achieved using three to four screws through the ilium [19].

### Rehabilitation protocol

Postoperatively, patients engaged in isometric whole‐body stabilisation exercises to strengthen the non‐operated leg. Post‐isometric relaxation techniques were applied to the operated leg. Controlled active‐assisted exercises were performed for the operated leg. Gait training was initiated on the day following the operation with 20 kg partial weight bearing and continued until the sixth week after surgery. For the first 6 weeks, specific movements such as forced adduction, external rotation, flexion over 90°, and active abduction from the lateral position were restricted. Immediately after the operation, patients were provided with a raised toilet seat and an arthrodesis seat cushion. X‐ray evaluations were taken in the sixth postoperative week. If signs of initial callus formation were observed, the weight‐bearing load was increased by 10–20 kg per week until full weight bearing was achieved, usually around 9–10 weeks after the operation.

### Protocol design and patient cohort

**Figure 1:**
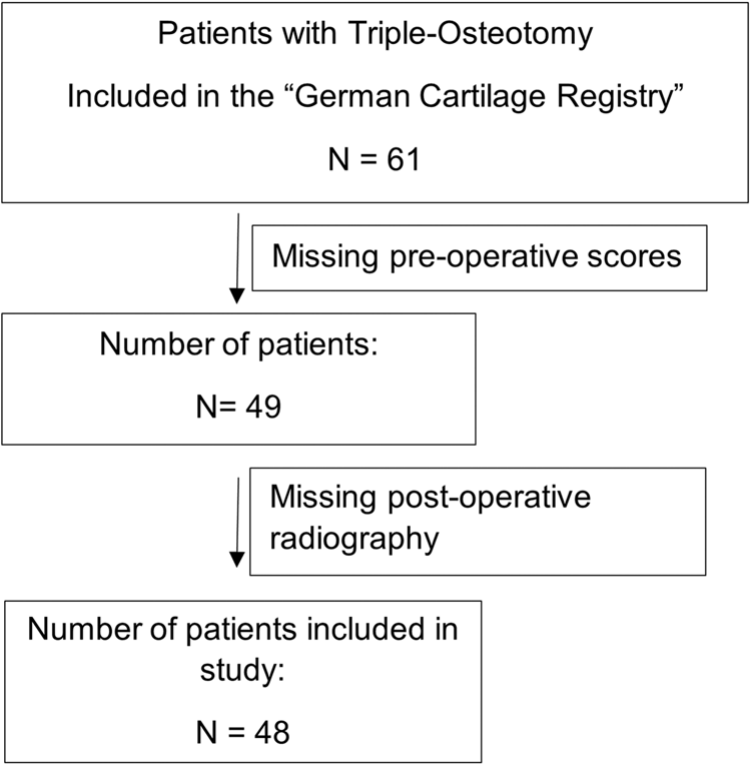
Patient flow chart.

All patients who underwent Triple‐Osteotomy at our medical center from 2015 onwards were invited to participate in the German Cartilage Registry (KnorpelRegister DGOU) (refer to Figure 1). Only patients without relevant joint degeneration were considered for surgery. Out of the total number of patients operated on, 61 patients were included in the registry. Patients without registered preoperative score data were excluded, resulting in 49 patients. One patient did not return for follow‐up radiography and was consequently excluded as well, leaving finally a total of 48 patients included in this study. Patients were invited for follow‐up examinations 6 weeks, 3 months, 6 months and yearly after the operation. The data input for the German Cartilage Registry and therefor this study was performed preoperatively and 6, 12, 24, 36 and 60 months after the operation. The follow‐up rate was 81.3% (39 of 48 patients) at 6 months, 77% (37 of 48 patients) at 12 months and 60.4% (29 of 48 patients) at 24 months with a mean follow‐up time of 24 months after 60 months. The time‐points 36 months and 60 months were not included in this study due to low follow‐up rates of 31.3% and 10.4%.

Regarding the collected data, the following parameters were assessed: age at the time of the operation, gender, smoking status, weight, height, number of previous hip surgeries prior to Triple‐Osteotomy and duration of symptoms before undergoing Triple‐Osteotomy (refer to Table 1).

**Table 1:** Patient demographics.

Parameter	Study group
Age (years)^a^	27.0 ± 7.5
Side (right)^b^	26 (54.2%)
Sex (male)^b^	5 (10.4%)
Smoker (yes)^b^	9 (18.8%)
Weight (kg)^a^	64.0 ± 11.1
Height (cm)^a^	166.5 ± 6.1
Hip operations prior to Triple‐Osteotomy (*n*)^b^	4 (8.3%)
Duration of symptoms prior to Triple‐Osteotomy (months)^a^	45.7 ± 44.3
Mean lateral centre‐edge angle (LCE)	16.56° ± 5.56°
Acetabular roof angle (HTE)	10.52° ± 5.23°
Vertical centre anterior angle (VCA)	17.48° ± 6.88°

a The values are presented as the mean ± standard deviation.

^b^ The values are given as the number of cases, with the percentage in parentheses.

Additionally, the questions outlined in Table 2 were evaluated using a scoring system. The Visual Analog Scale (VAS) was employed to rate pain levels on a scale of 1–100, with 1 representing the least and 100 indicating the worst pain. Patients were also asked about their ability to work due to hip pain.

**Table 2:** Questions supplementing the PRO scores regarding patient satisfaction and pain.

Question	Scoring
How did the treatment of your hip complaints help you overall?	1–5/Impairing – Very beneficial
Are you satisfied with the result of the operation?	1–5/Not satisfied – Very satisfied
How would you feel if you had to live with your current hip complaints for the rest of your life?	1–5/Not satisfied – Very satisfied
Do you currently still have pain in the operated joint?	1–4/Strong pain – No pain

Abbreviation: PRO, patient‐reported outcomes.

The iHOT33 with the subscores pain, function, sport, job‐ and social‐related aspects of daily life was utilised in this study [12]. The overall iHOT33 was obtained by summing the sub‐scores resulting in a score ranging from 0 to 100, with 0 reflecting the poorest outcome and 100 representing the best outcome. Our study evaluated intermediate‐term data, spanning from preoperative assessments to 24 months postoperatively.

The following cutoff values for the iHOT33 were applied based on the findings of the authors Ueland et al. [20]. The minimal clinically important difference (MCID) was defined as an improvement of >10 points. The substantial clinical benefit (SCB) was set at an improvement of >25 points between two‐time points. The absolute SCB at 24 months postoperatively was defined as an improvement of >64. The patient acceptable symptom state (PASS) was considered as a score of >58 points after 24 months.

### Radiographic analysis

**Figure 2:**
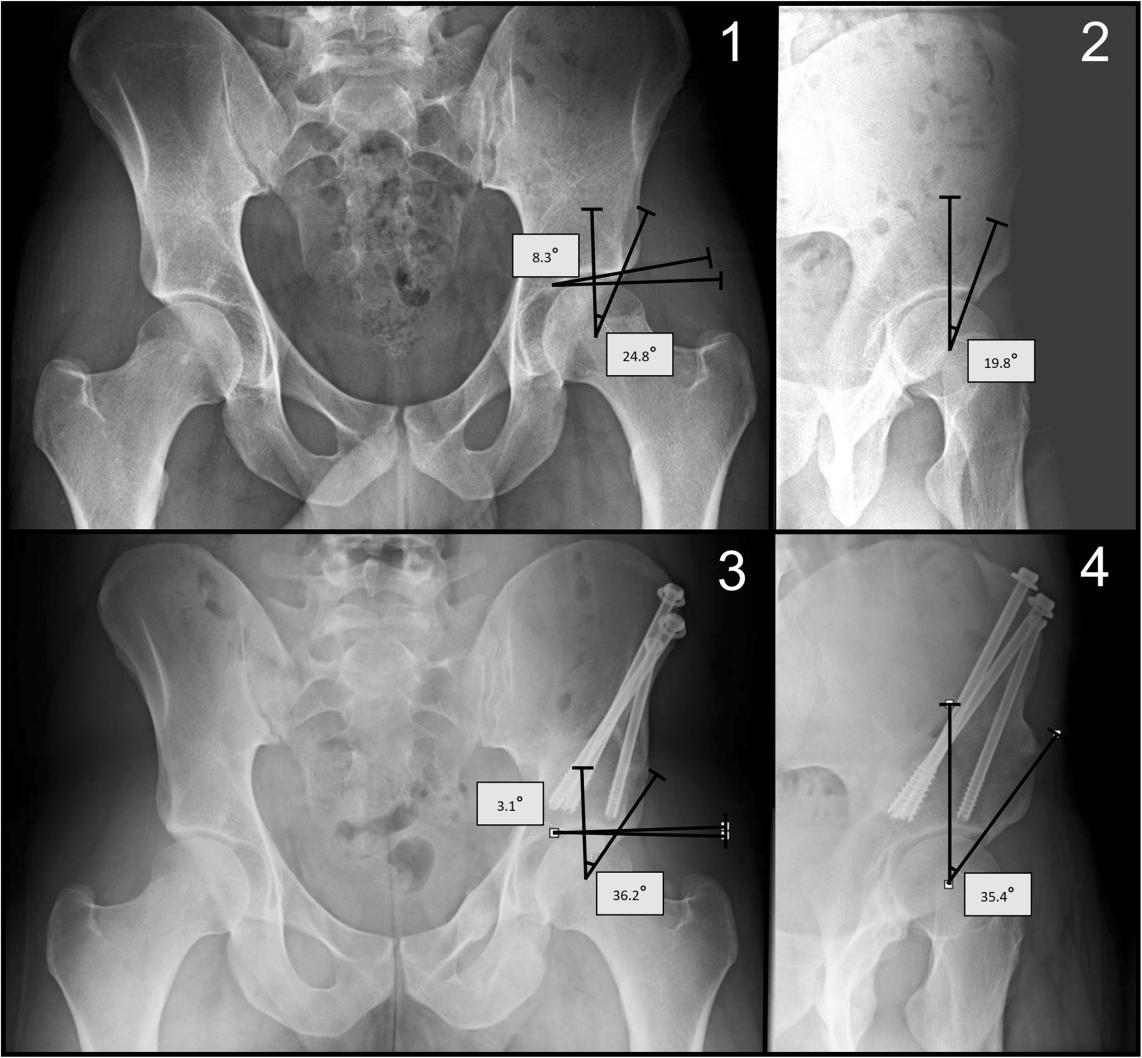
Examples of radiographical measurements. 1 and 3: Anterior‐posterior view of the pelvis, measurement of the “lateral center‐edge angle” (LCE angle) as well as the “acetabular roof angle” (HTE angle). 1 shows the preoperative measurement, 3 the postoperative measurement. 2 and 4: Faux‐profile projection with measurement of the “vertical‐center‐anterior angle” (VCA angle). 2 shows the preoperative measurement, 4 shows the postoperative measurement.

Radiographs obtained before and after the surgery were examined to evaluate the extent of the correction. The planning images for the procedure were utilised for preoperative angle calculations, and the initial postoperative images available were employed for postoperative angle calculations. The anterior‐posterior projection of the pelvis, taken in standing position, was utilised to determine the “lateral centre‐edge angle” (LCE angle) and the “acetabular roof angle/horizontal toit externe angle” (HTE angle) [1, 7, 9] (refer to Figure 2). The “vertical‐centre‐anterior angle” (VCA angle) was determined in the faux‐profile projection. Strict attention was paid to a standardised faux profile imaging. The respective image was taken in a standing position. The pelvis was rotated at an angle of 65° to the cassette. The foot was placed parallel to the film cassette and the leg to be examined was loaded with the body weight. The central beam was then directed at the femoral head.

### Statistical analysis

In cases where data was missing, the “last observation carried forward” method was applied. For the iHOT33‐Job sub‐scores, there were five patients who did not provide any response. In such instances, the worst possible outcome was assumed, resulting in a score of 0 for all time points. For patients with at least one response, the smallest possible change in outcome was presumed, meaning that the next available response was carried backward in time.

For paired values, such as scores at different time points, a paired *t*‐test was utilised. To determine the correlation between score values and independent variables, linear regression analysis was conducted including the variables age, side of operation, sex, smoker status, weight, height, number of operations prior to Triple‐Osteotomy, time point of Triple‐Osteotomy surgery, duration of symptoms prior to Triple‐Osteotomy as well as change in radiographic angles. The only exception was the responder analysis, where logistic regression analysis was employed. A significance level of *p* < 0.05 was considered statistically significant.

## RESULTS

**Figure 3:**
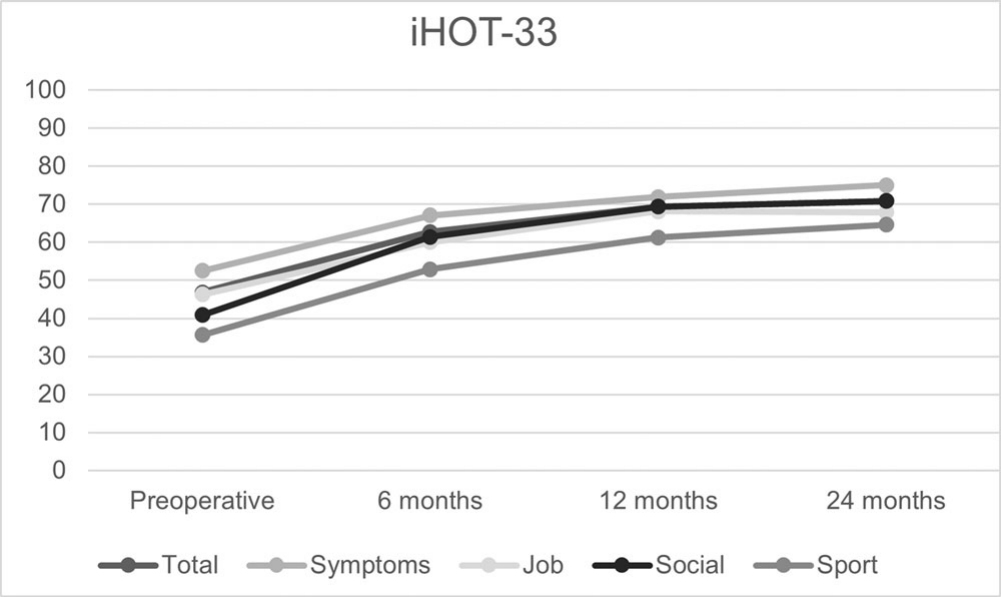
iHOT‐33 scores. All postoperative scores compared to the respective preoperative score showed significant results with a *p* < 0.01. iHOT‐33, International Hip Outcome Tool 33.

The iHOT‐33 total score and sub‐scores showed significant improvements at all postoperative time points compared to preoperative values (*p* < 0.01) (refer to Figure 3). The total iHOT‐33 score increased by 23.9 points at 24 months, with the largest gain (Δ 15.8) occurring within the first 6 months (refer to Table 3). Among sub‐scores, the social domain showed the highest improvement (Δ 30), while the sport sub‐score had the lowest baseline but improved by 29.0 points. The job and symptoms sub‐scores increased by 21.7 and 22.5 points, respectively.

**Table 3:** iHOT subscores and total score.

Category	Timepoint	Mean	Standard deviation	*p* value (paired *t*‐test)
iHOT JOB	Preoperative	46.2	32.46	
				0.001*
	6 months	60.0	31.35	
				<0.001*
	12 months	68.1	31.26	
				0.471
	24 months	67.9	32.02	
iHOT social	Preoperative	40.8	21.22	
				<0.001*
	6 months	61.5	26.97	
				<0.001*
	12 months	69.4	27.16	
				0.234
	24 months	70.8	25.75	
iHOT sport	Preoperative	35.6	23.78	
				<0.001*
	6 months	52.9	26.81	
				0.003*
	12 months	61.3	27.80	
				0.097
	24 months	64.6	25.60	
iHOT symptoms	Preoperative	52.5	19.62	
				<0.001*
	6 months	67.1	23.06	
				0.004*
	12 months	71.9	22.85	
				0.058
	24 months	75.0	21.73	
iHOT total	Preoperative	46.9	17.76	
				<0.001*
	6 months	62.7	22.19	
				<0.001*
	12 months	69.4	22.73	
				0.224
	24 months	70.8	20.71	

*Note*: *p* value is given as paired *t*‐test. When compared to the respective preoperative value, all postoperative scores are significantly altered (*p* < 0.001).

Abbreviation: iHOT, International Hip Outcome Tool.

**Figure 4:**
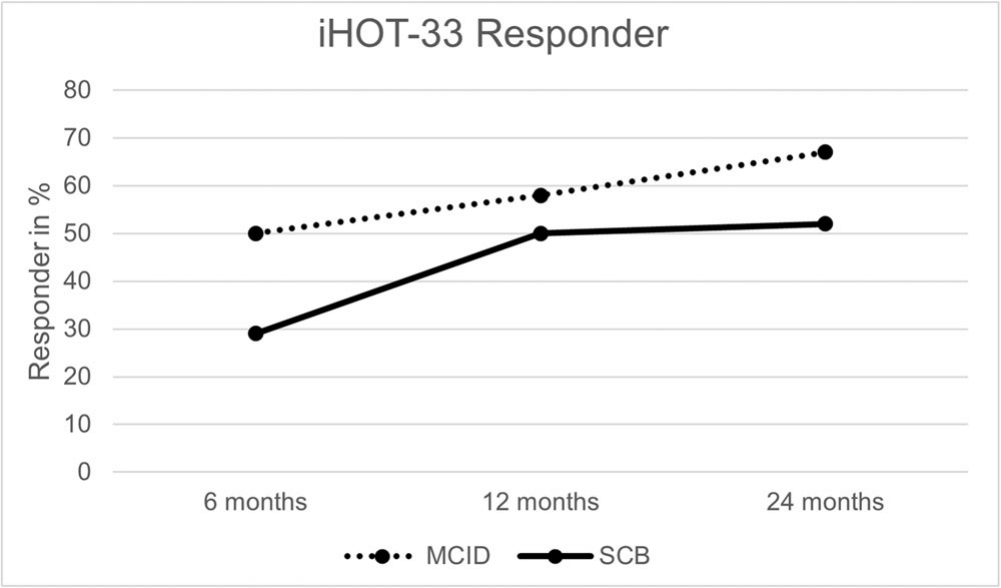
iHOT‐33 Responder Rates in %. MCID = Minimal clinically important difference, measured between the baseline preoperatively and the respective month. Responders were set to a difference of >1 point. SCB = Substantial clinical benefit, measured between the baseline preoperatively and the respective month. Responders were set to a difference of >2.5 points. iHOT‐33, International Hip Outcome Tool 33.

By 6, 12 and 24 months postoperatively, 50%, 58.3% and 66.7% of patients achieved the MCID, respectively (refer to Figure 4). SCB was observed in 29.2% of patients at 6 months, 50% at 12 months and 52.1% at 24 months. Furthermore, 68.8% of patients achieved absolute SCB at 24 months, and 79.2% met the PASS.

**Figure 5:**
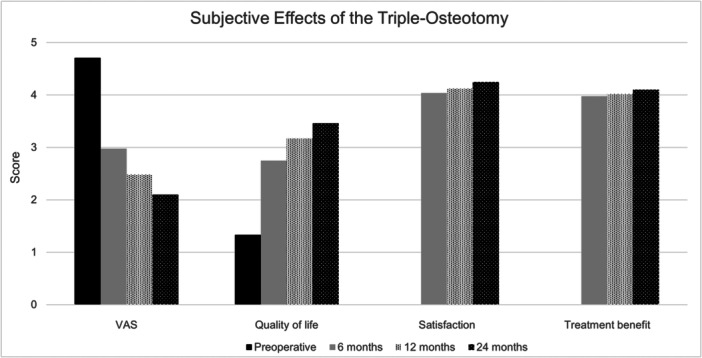
Subjective effects of Triple‐Osteotomy.

Pain levels, measured by the VAS, decreased from 4.7 ± 2.6 preoperatively to 2.1 ± 2.3 at 24 months, with reductions at all interim time points (3.0 at 6 months and 2.5 at 12 months) (refer to Figure 5). Quality‐of‐life scores increased from a mean of 1.33 ± 0.7 preoperatively to 3.5 ± 1.6 at 24 months, with statistically significant changes at all intervals. Satisfaction scores increased from 4.0 ± 1.15 at 6 months to 4.2 ± 1.1 at 24 months, while treatment benefit scores rose slightly from 3.98 ± 1.02 at 6 months to 4.10 ± 0.95 at 24 months. Regarding employment, four patients were unemployed due to hip pain before surgery, but none remained unemployed for this reason at 24 months.

**Figure 6:**
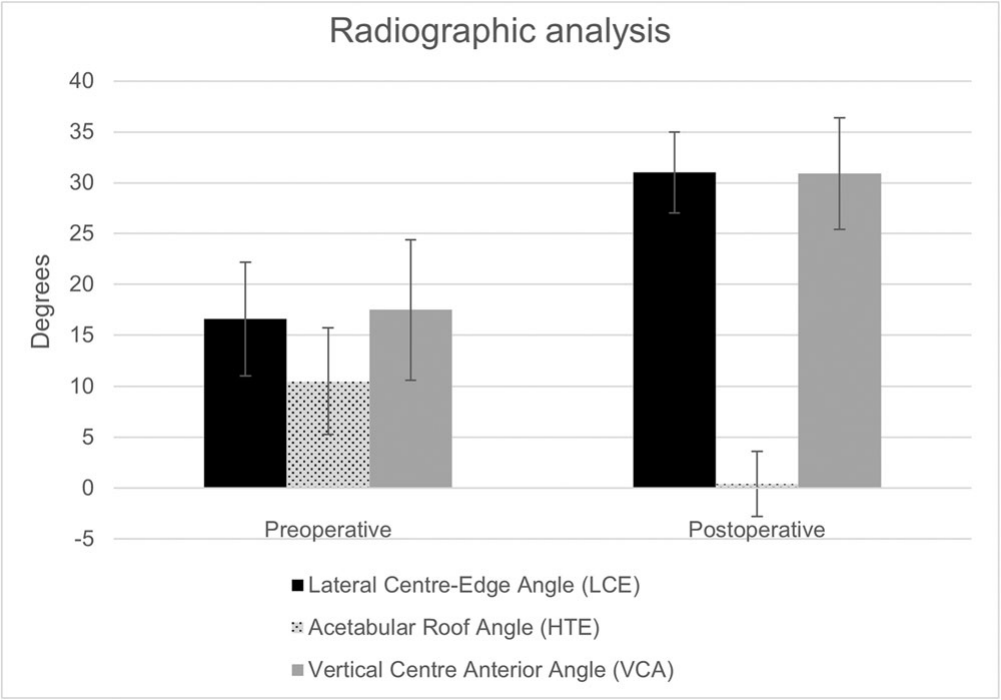
Radiographic analysis.

Radiographic parameters showed improvement, with the lateral center‐edge angle increasing from 16.56° ± 5.56° to 30.96° ± 3.96° and the acetabular roof angle decreasing from 10.52° ± 5.23° to 0.44° ± 3.20° (refer to Figure 6).

Regression analysis found no significant correlation between radiographic changes and iHOT‐33 scores. Pain relief, functional improvement and changes in quality of life were observed over 24 months.

## DISCUSSION

This study analysed the mid‐term results of Triple‐Osteotomy in 48 patients using the iHOT33. The main findings of this study demonstrate that Triple‐Osteotomy significantly improves patient‐reported outcomes, particularly in social and sports domains, as measured by iHOT33. These improvements, with a mean increase of 23.9 points in total iHOT33 scores, underscore the substantial impact of Triple‐Osteotomy on patients' quality of life.

The most significant gains occurred within the first six months postoperatively (Δ 15.8), emphasising the procedure's rapid benefits for pain relief and functional recovery. Importantly, the social domain exhibited the greatest improvement (Δ 30), reflecting the procedure's ability to restore patients' ability to engage in meaningful social and recreational activities. These results confirm that Triple‐Osteotomy is a highly effective hip‐preserving surgery, particularly for younger, active patients with symptomatic hip dysplasia.

These findings are consistent with the results observed in a study conducted by Wu et al. [22], where they also reported significant improvements in patients using the Harris Hip Score as the primary outcome measure. Another study by Von Bremen‐Kühne et al. [21] demonstrated a notable increase in the HHS score, from an average of 52.4 points to 81.1 points after an average follow‐up of 42.7 months. Similarly, Ettinger et al. [4] found significant improvement in the HHS score, with scores changing from 63.3 preoperatively to 90.1 at the time of follow‐up. Comparable results were reported by Janssen et al. [10], who observed an increase in HHS scores from 70.2 to 81.2 points after an average follow‐up period of 11.5 years. The HHS score shows the significant improvement after multidimensional axis correction, but does not reflect the specific needs of young, athletic patients.

The iHOT‐33 sub‐scores demonstrated significant improvements at 24 months postoperatively, with the social sub‐score showing the largest increase, highlighting the surgery's positive impact on patients' ability to engage in social and daily activities. In contrast, the sport sub‐score started from the lowest baseline, reflecting the greater challenges in restoring high‐demand athletic functions. Subjective quality of life scores also showed continuous and substantial improvement. Notably, improvements were significant at all assessed time points, suggesting progressive adaptation of the surrounding hip musculature to the corrected anatomical configuration over time.

When compared to PAO, the outcomes of Triple‐Osteotomy exhibit both similarities and potential advantages. Like PAO, Triple‐Osteotomy improves femoral head coverage and quality of life, as evidenced by previous studies using PROMs such as the Harris Hip Score (HHS) [2, 5, 16, 18]. However, the early and substantial improvements observed in this study may differentiate Triple‐Osteotomy from PAO, which often requires longer recovery periods. This distinction could be attributable to differences in surgical technique. While PAO involves a more extensive capsulotomy and acetabular reorientation, Triple‐Osteotomy achieves similar biomechanical corrections with a focus on three‐dimensional acetabular positioning. The relative preservation of soft tissue structures in Triple‐Osteotomy may facilitate faster periarticular muscle adaptation and pain reduction, contributing to the observed early recovery.

Despite the observed benefits, radiographic improvements in lateral center‐edge and acetabular roof angles showed no correlation with iHOT33 scores. This finding aligns with previous literature suggesting that clinical outcomes depend not only on anatomical correction but also on patient‐specific factors, such as preoperative activity levels and psychosocial support [11, 17]. Thus, the integration of PROMs with radiographic measures is essential for a holistic evaluation of surgical success.

## LIMITATIONS

This study has several limitations that warrant discussion. First, the retrospective design and single‐center setting introduce potential selection and reporting biases, which may limit the generalisability of the findings. The lack of a control group, such as patients undergoing alternative procedures like PAO, restricts direct comparisons of outcomes across hip‐preserving surgeries. Radiographic assessments performed by a single observer, while ensuring consistency, raise concerns regarding interobserver reliability.

Although attention was paid to standardised faux profile recording, patients' pelvic tilting cannot be completely ruled out. As Putnam et al. [4] showed in a cadaver study, an increased tilt of 10° can increase the measured VCA by 6°. Although this error is probably uniform across the number of patients, it must be taken into account for follow‐up studies [14].

The hip angles were quantified by a sole individual, ensuring uniform measurement and comparability of the angles. Nonetheless, the absence of multiple measurements or assessments by a second individual represents a potential study limitation. However, Nepple et al. [13] have shown excellent intra‐ and interobserver reliability of the radiographic analysis of hip dysplasia, specifically having examined the LCE and HTE as used in our study. Therefore, we remain assured that our study results retain significance despite this limitation.

Variability in patient adherence to the structured rehabilitation protocol may have also impacted functional recovery and PROM improvements. Preoperative differences in physical fitness and activity levels likely introduced additional confounding variables. Lastly, the follow‐up rate of 60.4% at 24 months, while acceptable, could have resulted in selection bias, as patients with less favourable outcomes may have been less likely to participate. The sample size of 48 patients, though sufficient for initial conclusions, may limit the statistical power to detect subtle differences or correlations, particularly in subgroup analyses. Future studies should employ prospective, multicentre designs with larger sample sizes and control groups to validate these findings. Additionally, standardised radiographic imaging protocols and multiple observer analyses would improve reliability, while exploring long‐term PROM sustainability beyond 24 months would provide deeper insights into the benefits of Triple‐Osteotomy.

## CLINICAL IMPLICATIONS AND FUTURE DIRECTIONS

The findings of this study suggest that Triple‐Osteotomy is a highly effective option for improving quality of life in patients with hip dysplasia. While the outcomes are comparable to PAO, the early improvements observed with Triple‐Osteotomy may make it a preferred choice in cases where prompt functional restoration is a priority. Future studies should directly compare Triple‐Osteotomy and PAO, with a focus on understanding the biomechanical and rehabilitative factors driving these differences. Additionally, long‐term follow‐up is needed to assess whether the early advantages of Triple‐Osteotomy translate into sustained benefits over time.

## CONCLUSION

Triple‐Osteotomy offers significant and rapid improvements in patient‐reported outcomes for individuals with hip dysplasia, particularly in enhancing social and sports‐related quality of life as measured by iHOT33 and other subjective assessments. Its potential advantages over Periacetabular‐Osteotomy, especially in terms of early recovery, warrant further investigation through prospective, comparative studies to better define its role in hip‐preserving surgical strategies.

## AUTHOR CONTRIBUTIONS

*Conceptualization*: Julia Elisabeth Lenz and Stefan Fickert. *Methodology*: Julia Elisabeth Lenz. *Formal analysis and investigation*: Julia Elisabeth Lenz. *Writing–original draft preparation*: Julia Elisabeth Lenz and Stefan Fickert. *Writing–review and editing*: Moritz Riedl, Dominik Szymski, Stefan Landgraeber, and Volker Alt. *Supervision*: Stefan Fickert and Stefan Landgraeber.

## CONFLICT OF INTEREST STATEMENT

The authors declare no conflicts of interest.

## ETHICS STATEMENT

None declared.

## Data Availability

Data and statistical analysis are available from the authors upon reasonable request.
